# Exploring health care providers’ experiences of and perceptions towards the use of misoprostol for management of second trimester incomplete abortion in Central Uganda

**DOI:** 10.1371/journal.pone.0268812

**Published:** 2022-05-19

**Authors:** Susan Atuhairwe, Kristina Gemzell-Danielsson, Lynn Atuyambe, Josaphat Byamugisha, Nazarius Mbona Tumwesigye, Claudia Hanson

**Affiliations:** 1 Department of Obstetrics and Gynaecology, Makerere University, Kampala, Uganda; 2 Department of Reproductive Endocrinology and Infertility, Mulago Specialised Women and Neonatal Hospital, Kampala, Uganda; 3 Department of Women´s and Children´s Health, Karolinska Institutet, Stockholm, Sweden; 4 Department of Community Health and Behavioural Sciences, Makerere University School of Public Health, Kampala, Uganda; 5 Department of Epidemiology & Biostatistics, School of Public Health, Makerere University, Kampala, Uganda; 6 Global Public Health, Karolinska Institutet, Stockholm, Sweden; 7 Department of disease control, London School of Hygiene and Tropical Medicine, London, United Kingdom; Flinders University, AUSTRALIA

## Abstract

**Introduction:**

Women living in low- and middle-income countries still have limited access to quality second trimester post abortion care. We aim to explore health care providers’ experiences of and perceptions towards the use of misoprostol for management of second trimester incomplete abortion.

**Methods:**

This qualitative study used the phenomenology approach. We conducted 48 in-depth interviews for doctors and midwives at 14 public health facilities in central Uganda using a flexible interview guide. We used inductive content analysis and made code frequencies based on health care provider cadre, and health facility level and then abstracted themes from categories.

**Results:**

Well trained midwives were perceived as competent to manage second trimester post abortion care stable patients, however doctor’s supervision in case of complications was considered important. Sometimes, midwives were seen as offering better care than doctors given their stronger presence in the facilities. Misoprostol received unanimous support and viewed as: safe, effective, cheap, convenient, readily available, maintained patient privacy, and saved resources. Challenges faced included: side effects, prolonged hospital stay, treatment failure, inclination to surgical evacuation, heavy work load, inadequate space, lack of medical commodities, frequent staff rotations which affects the quality of patient care. To address these challenges, respondents coped by: giving patients psychological support, analgesics, close patient monitoring, staff mentorship, commitment to work, team work and patient involvement in care.

**Conclusion:**

Misoprostol is perceived as an ideal uterine evacuation method for second trimester post abortion care of uncomplicated patients and trained midwives are considered competent managing these patients in a health facility setting with a back-up of a doctor. Health care providers require institutional and policy environment support for improved service delivery.

## Introduction

Globally, an estimated 23 000 women die annually from complications of unsafe abortion [1]. Over 97% of unsafe abortions occur in low- and middle-income countries (LMICs) where abortion laws are very restrictive [2]. LMICs often have over-stretched health systems, huge patient caseloads, scarcity of health care providers (HCPs), stigma, lack of essential drugs and supplies, low quality care, and inadequate access to post abortion care (PAC) services [3–5]. There is a complex interplay between these factors. Women with unsafe abortions often present with complications that need hospitalization [2]. This huge patient caseload from abortion morbidity requires resources like trained and competent health care providers (HCPs), drugs and commodities, and health financing amongst others [6]. In settings with inadequate human resource and frequent stock outs of essential drugs and supplies [7], this huge caseload over-stretches the health system and inadvertently reduces the quality and access to care [8]. Even when the HCPs are available, some women may still get low quality care due to abortion stigma [9]. In the community, abortion stigma may deter women from accessing care at public health facilities [10], yet they may not afford the private facilities which charge premium rates to treat women abortion complications. These women therefore delay to receive care and have more severe complications when they reach the facilities that require more resources putting an even greater burden on the already over-stretched system. Despite the restrictive abortion laws, PAC is accepted in nearly all countries [11]. Evidence from The International Federation of Gynecology and Obstetrics across 46 countries in seven regions, shows that improvements in the competences of HCPs to increase the spectrum of PAC services they can provide improves access to quality PAC [4].

PAC is a package of five key life-saving interventions for patients with abortion complications [12]: 1) emergency treatment of abortion complications, 2) family planning, 3) counseling, 4) other reproductive and related health services, and 5) community–service provider partnerships [13]. HCPs are central in the provision of PAC services both at the health facility and community levels [12]. National policy guidelines are to inform and guide HCPs to safeguard standards in service provision. However, translation of policy into action at the facility level is influenced by a number of factors including attributes of the HCPs for example their knowledge, skills, motivation, and confidence in performing new tasks [14,15].

There is consensus internationally that both physicians and non-physicians are adequately trained HCPs to manage first trimester abortion complications using misoprostol or MVA [3,12,16,17]. Inclusion of midwives in PAC provision is a recognized high impact practice that improves access to care [18]. Even in countries, like Nigeria, Uganda, Kenya, and Rwanda with restrictive abortion laws, midwives are recognized and highly acceptable HCPs for first trimester PAC offering a variety of services like screening, counselling, uterine evacuation, and family planning provision [3]. In contrast, reservations persist regarding whether or not non-physician HCPs can safely provide PAC in the second trimester [19]. In view, that physicians are too few in numbers, and typically unequally distributed in most LMICs, creating evidence if non-physicians can equally safely and effectively handle second trimester as first trimester abortion is critical. However, such interventions also need the support of HCPs, and their views on task sharing are essential to augment provision of quality health care [20].

### Uganda in context

The total fertility rate in Uganda is estimated at 5.0 births per woman, which currently is the eleventh highest in the world [21]. Over 75% of Uganda’s is below 30 years, and modern contraception utilization remains low at 35% in married women [22]. The unmet need for contraception is estimated at 28% [22], and approximately half of the unintended pregnancies occur because women do not use contraception [23]. Induced abortion is controversial and restricted in Uganda and legally permitted only to save a woman’s life [24]. As a result, women often resort to unsafe abortion- that’s either performed by a person lacking the necessary skills or in an environment that does not conform to minimal medical standards [12]. Annually 12 per 1,000 women aged 15–49 years are hospitalized for induced abortion complications [23], which is high compared to other countries in Sub-Saharan Africa like Nigeria (6 per 1000 women) and Rwanda (7 per 1000 women) [25,26].

In Uganda, key elements of PAC services are provided from health centre level up to referral hospitals according to ministry of health policy guidelines [27]. Health centres II and III provide basic emergency obstetric care while health centre IVs and hospitals provide comprehensive emergency obstetric care services. Heath centre IVs and general hospitals are considered secondary care facilities and referral hospital provide tertiary care services. While there are midwives at all levels of health care, doctors are only present at secondary and tertiary level health care facilities. However, there are gaps in service delivery; ranging from a lack of adequate skilled workforce, infrastructure, drugs, supplies and health financing [5,28]. Midwives are the primary HCPs of PAC including removal of retained products using MVA, counseling and provision of family planning at the majority of health facilities [7]. Doctors are usually only called in to review patients with severe complications or when a surgical procedure is required. Midwives assure continuity of care as they spend their full shifts on the ward when on duty, which gives them more time to interact with patients, and provide more PAC related information.

Misoprostol was introduced for PAC at Ugandan public health facilities first in 2009, as part of a pilot project [29], and later taken to scale 2011 [27]. Misoprostol provides patients and HCPs an increased choice of uterine evacuation methods [12]. Both physicians and midwives, consider misoprostol acceptable for treatment of first trimester abortion complications [30]. Curettage for surgical evacuation of retained products of conception, though considered outdated by the World Health Organization (WHO) due to the associated risks for complications, is still practiced in Uganda as the preferred treatment modality for second trimester PAC, and exclusively performed by physicians [27].

Within this background, we conducted a multi-centre randomized controlled trial (RCT) in Uganda to evaluate the safety, effectiveness and acceptability when misoprostol treatment is administered by either midwives or physicians to women with second trimester incomplete abortion [31]. To understand the feasibility and acceptability of the intervention, this paper aims to explore HCPs’ experiences of and perceptions towards the use of misoprostol for management of second trimester incomplete abortion. Gaining a deeper understanding of the HCPs’ perspective of this phenomenon gives insights on possible policy implementation hurdles and how these can be addressed beforehand.

## Materials and methods

### Study design

This qualitative study, using in-depth interviews, was a sub-study to the RCT and carried out on HCPs actively participating in the RCT for at least one year [31]. We found phenomenology, the most appropriate methodology, to gain deeper insights in the HCPs’ lived experiences of managing patients participating in the RCT [32]. Phenomenology is a philosophy of experience; therefore, the commonality of the HCPs’ lived experiences provides a composite description of the phenomenon while bracketing the researcher’s preconceived assumptions about the phenomenon [33]. We used the checklist for consolidated criteria for reporting qualitative research as a guide for data presentation and reporting [34].

### Participant selection

We purposively sampled doctors and midwives working on the maternity or gynaecology wards and actively participating in the RCT. The research team made regular bimonthly visits to the study sites and as such could identify HCPs that were able to participate in the interviews. We generated a sampling frame to have sufficient participation from referral level facilities and a good balance between doctors and midwives, although few doctors were working in the included facilities. Based on the sampling frame, the research team generated a list of HCPs with their telephone contacts and study sites, and handed this over to the interviewers for scheduling of appointments. The interviewers contacted the HCPs on telephone, all agreed to participate and interviews were conducted as scheduled, except for two doctors who postponed, due to their busy timetable.

We recruited participants between June and July 2020 until the point of saturation and by then had 16 doctors and 32 midwives. The doctors included medical officers, obstetrics and gynaecology residents, and obstetricians and gynaecologists. The midwives’ levels of professional training ranged from enrolled midwife (certificate qualification), registered midwife (diploma qualification), and degree level. Some of the respondents were nurses who were either double trained as midwives or had practiced midwifery for a long time and were actively working in maternity or gynaecology wards.

We used in-depth interviews to capture information on HCPs’ experiences and perceptions on use of misoprostol in second trimester. In-depth interviews provide more comprehensive information on a subject matter [35]. The privacy in-depth interviews offer, allow HCPs to freely share information particularly on sensitive issues that affect service delivery at their work place [36].

### Setting

Respondents were sampled from 14 public health facilities in Central Uganda, which comprised of two referral hospitals, eight general hospitals and four health centre IVs in urban, rural and peri-urban areas, see “Table 1”. The health centre IV is the first level health facility to offer comprehensive emergency obstetric care service in Uganda. Referral is then made to the general hospital and finally the referral hospitals.

**Table 1:** Characteristics of participating public health facilities.

District	Health facility level	Location	Average monthly post abortion care caseload-2019*	Number of health care providers	
				Physicians	Midwives
Buikwe	General Hospital	Rural	13	6	15
Gombe	General Hospital	Rural	21	7	14
Kassanda	Health centre IV	Rural	12	2	8
Kayunga	General Hospital	Rural	20	4	12
Luweero	General Hospital	Rural	18	4	9
Masaka	Referral Hospital	Rural	17	3	21
Mityana	General Hospital	Urban	20	7	17
Mpigi	Health centre IV	Rural	12	3	16
Mukono	General Hospital	Urban	17	3	16
Nakaseke	General Hospital	Rural	13	6	20
Wakiso	Health centre IV (2)	Peri-urban	15	2	5
	General Hospital	Urban	20	4	25
	National Referral Hospital	Urban	35	10	10

*Source—Health facility records.

We interviewed the participants at the health facilities in a private area, usually the boardroom, compound or the ward In-charge’s office. We gave each participant a unique appointment time so that we could interview one participant at a time. No other staff member was present at the time of the interview. The exception was participants for one study site that was converted to a covid-19 treatment center. We interviewed these participants at places of their convenience but still ensured that privacy was maintained.

### Data collection and quality assurance

We used a pilot tested interviewer guide with open-ended questions and probes providing flexibility in English language S1. The interview guide allowed flexibility and we made adjustments to the guide as appropriate based on the depth of emerging ideas. Topics covered included: perceptions and experiences with uterine evacuation methods offered in second trimester, use of misoprostol for second trimester PAC, how the work environment affects service provision, and how midwives deliver second trimester PAC using misoprostol compared to doctors.

Interviews were carried out by two research assistants who were trained and supervised by SA. At each appointment, the research assistants began by explaining the study to the HCP highlighting the rationale, benefits, risks, and rights to withdraw from the study. When the HCP agreed to participant, each respondent then signed an informed written consent. Each interview lasted approximately forty to fifty minutes and participants received a transport reimbursement. We did not conduct any repeat interviews. We tape recorded all interviews, and the scribe took notes during the interviews. We held daily debrief meetings where we discussed emerging ideas, and this guided our decision for meaning saturation when there was no new information to answer our research question. In addition, extensive field notes of experiences in the field were written together with the daily debrief meeting notes. We did not conduct participant observations. We transcribed all the data and the principal investigator read through all transcripts to ensure completeness of the data. None of the transcripts was returned to the participants for comment or correction.

We obtained ethical clearance from Makerere University School of Medicine Research and Ethics Committee—Rec ref 2017–016 and Uganda National Council for Science and Technology—HS153ES. All participating health facilities gave administrative clearances and study participants gave informed written consent. Data was anonymized by assigning study participant unique identification numbers. We adhered to the Covid-19 guidelines released by the ministry of health [37].

### Data analysis

We used inductive content analysis, given that the research area (on second trimester PAC task sharing) was new and we were not conducting any theory testing [32]. Transcribed text was read through several times to obtain a sense of the whole. The unit of analysis was taken as a whole individual interview to preserve the context. The main researcher identified the content areas that addressed the research question. By inductive approach, the text was divided into meaning units, condensed and labelled with a code, using HyperRESEARCH software version 4.5.0, to constitute the manifest content. The abstracted codes were grouped, and formed primary and secondary categorizes using an iterative process. A second coder reviewed the codes and categories made, and discussions were held to obtain agreement between the two coders. We formed a coding sheet reflecting code frequencies for each HCP cadre (doctor or midwife) and health facility level (referral hospital, general hospital and health centre IV) to ease interpretation of results at each of those levels. An interpretation of the underlying meaning (latent content) was generated through discussion to abstract themes from the categories. These themes were presented to the HCPs for clarification and validation of their true meaning. A review of the literature and further reflection was done to further consolidate the themes.

## Results

We interviewed 32 midwives and 16 doctors, S1 Table. The average age of participants was 40 (range 21 to 59) and 38 (range 28 to 60) years for midwives and doctors respectively. Midwives had an average of 15 (range two to 30) years in clinical practice, and the doctors 11 (range one to 39) years. Both midwives and doctors had an average of eight years in PAC practice with an upper range value of 30 and 20 years, respectively. Four doctors, practiced at the referral hospitals, eight at the general hospitals and four at health centre IVs. Among the midwives, eight practiced at the referral hospitals, 16 at the general hospitals and eight at health centre IVs.

**Fig 1:**
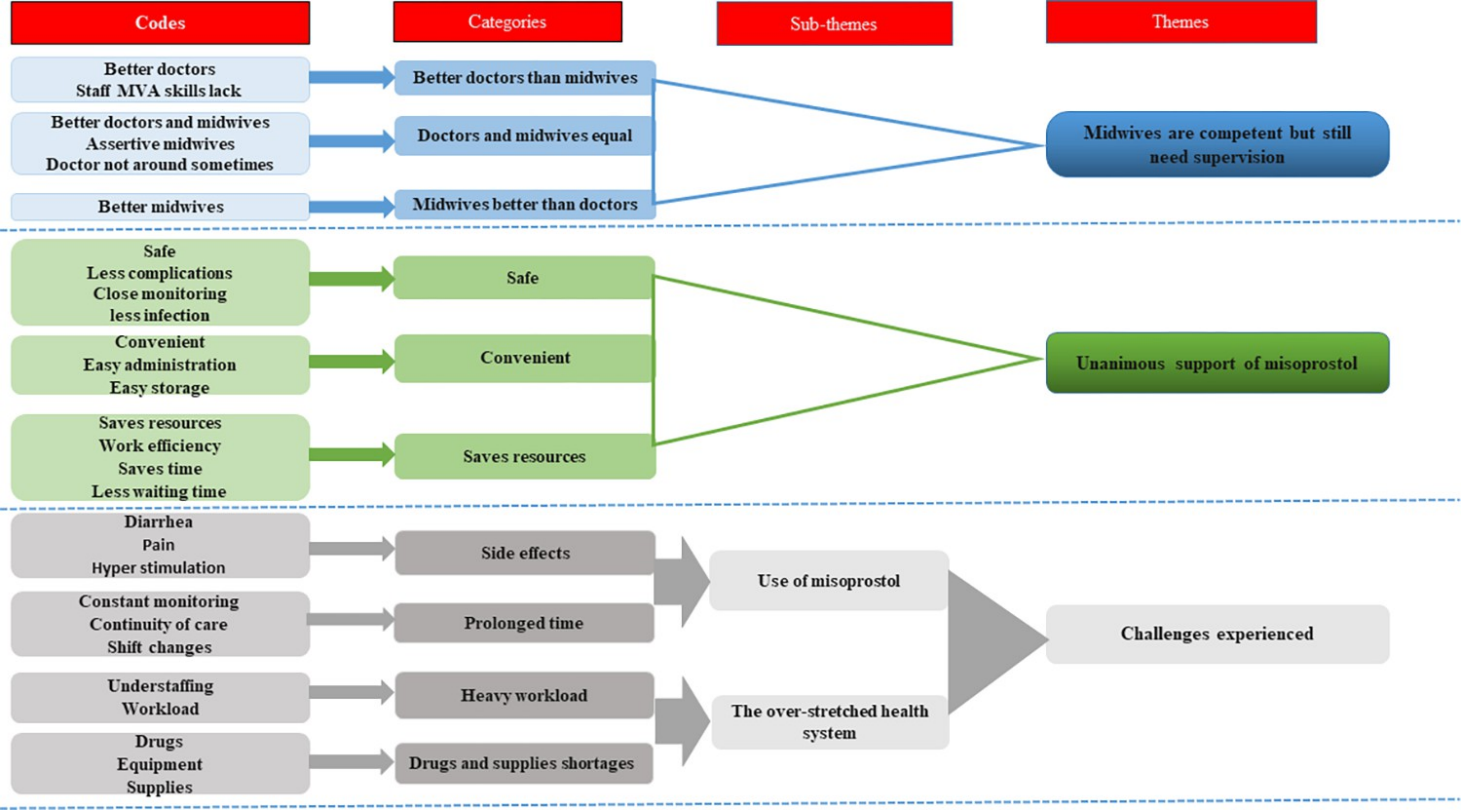
An example of the abstraction process- generation of themes from the codes. MVA–Manual vacuum aspiration.

Analysis derived three themes from the qualitative data: i) midwives are competent but still need supervision, ii) unanimous support of misoprostol, and iii) challenges experienced emerging from the use of misoprostol and the overstretched health system, (Fig 1).

### Midwives are competent but still need supervision

Participants indicated that once HCPs receive training, perform appropriate patient assessment, and follow the treatment guidelines, then medical management is easy and should be the same irrespective of the health cadre with good outcomes. At all facilities, majority of the doctors and some midwives echoed that midwives can provide medical management of second trimester PAC as effectively as doctors:

*I think they [midwives] can because there is not much needed*, *if they [midwives] are taught the right doses*, *the frequencies and what to look out for and dangers and tell them what to do when they get challenges*. *I think it is ok because there is nothing hard*. *(participant 47_doctor_referral hospital)*

Some midwives at all health facility levels and a few doctors at the hospitals felt that midwives could provide better care than doctors because of: i) availability, ii) commitment, iii) experience, and iv) confidence in providing PAC:

*Yeah*, *they [midwives] are always available and these mothers are always in their hands*. *Yes*. *They have experience*, *and they have that confidence*, *they have worked on many*. *But our physicians; they are dealing with operation and those things*. *So*, *yeah*, *but they [midwives] are better than doctors I am telling you*. *(participant 9_midwife_health centre)*

Only one doctor and few midwives at general hospitals and health centre IVs cautioned that midwives needed a back-up of a medical doctor in case of a complication requiring surgical intervention. Sometimes doctors preferred being informed of patients treated by midwives, and a few doctors at general hospital level echoed that midwives were uncomfortable treating second trimester PAC patients alone due to fear. This team work synergizes the doctors and midwives’ efforts in patient care and also improves the midwives’ self-assurance as illustrated below:

*Yeah*, *they [midwives] can but there is that fear that what if this patient gets this [complication]*. *… So*, *they can always handle provided the medical officer is also there*, *so*, *that in case of a complication*, *they can easily consult*. *But if they are alone somewhere*, *they feel not so comfortable*. *But at least it just needs time to build confidence in them so that they can also do it*. *(participant 42_doctor_general hospital)*

### Unanimous support of misoprostol

Respondents at all health facility levels perceived misoprostol as very safe for second trimester PAC, if used by a skilled HCP on a clinically stable patient. However, they asserted that patients should be closely monitored in a health facility setting, for prompt intervention in case of profuse vaginal bleeding. HCPs cited that patients treated with misoprostol experienced less pain, trauma, and infection compared to surgical evacuation. Few patients experienced side effects, which were manageable, and patients were satisfied with the treatment:

*The risks of trauma to the mother are so much lessened*, *…*, *you would avoid so many other complications that come from the use of instruments trying to evacuate*. *So*, *most of the mothers that have been counselled on that method have accepted genuinely*. *(participant 35_doctor_referral hospital)*

Nearly all respondents described misoprostol as very effective and majority of patients get a complete abortion even before completion of all the repeated misoprostol doses. Misoprostol was perceived as fast acting although not as fast as the evacuation or MVA. Notably, misoprostol success rates are high if used according to the dosage schedule and proper patient assessment made prior to treatment initiation. A few HCPs conveyed that misoprostol appeared to work better in second trimester compared to first trimester. Some HCPs were surprised at how effective misoprostol was and had grown confidence in using it:

…. *Actually*, *to me I got two failures that I had to go for Curate [surgical evacuation] but other than that what I can say roughly before even you reach the 5th dose when you go to assess the mother you find it that is actually very effective*. *… I was so shocked seriously*, *I was surprised*. *(participant 22_doctor_general hospital)*

Misoprostol was also considered easy to administer, convenient, cheap, easy to store, maintained patient privacy, and acceptable by patients and HCPs. HCPs mainly at hospital level mentioned that using misoprostol saves a lot of resources in terms of patient waiting time and, need for surgical instruments and theatre facilities. It improves work efficiency by allowing the few staff to multitask:

*Me I think the use of misoprostol is the best because it is easy to use*… . *Sometimes in these village hospitals you might look for a doctor to do an evacuation the mother is bleeding …*, *but if you start the misoprostol*, *the good thing with it*, *in most patients they respond*. *So*, *you might continue with your other things as you monitor this mother*. *(participant 6_midwife_general hospital)*

Midwives particularly at hospital level highlighted additional benefits of misoprostol of being readily available at the health facilities because it is included in the country’s essential drug list. Misoprostol may improve future pregnancy outcomes given that there is no direct physical insult made to the uterus. Patients are actively involved in their management which improves their psychological health status:

*The mothers don’t feel comfortable undressing unless this mother is in shock*. *But these tablets are comfortably taken*. *And they will tell you that I prefer taking tablets than you undressing me and putting those metals in me*. *So*, *that is the advantage of using misoprostol*. *(participant 29_midwife_general hospital)*

### Challenges experienced

This theme emerged from two subthemes on: challenges of using misoprostol and the overstretched health system.

#### Challenges of using misoprostol for second trimester PAC

The most prominent challenge cited by HCPs at all health facility levels was side effects experienced by patients like; shivering, nausea, vomiting, diarrhea, fever, and abdominal cramps as illustrated below:

*We counsel the mother before administering miso because she may experience rigors*, *abdominal pain; especially rigors or shivering after taking misoprostol*. *(participant 33_midwife_referral hospital)*

Patients using misoprostol were required to stay in the health facility almost 24 hours for close monitoring. This challenge of prolonged treatment time seemed more evident among the adolescents and women with induced abortions. These patients usually want to exit health facilities faster because they do not want to be identified by their relatives or friends as having abortion-related complications:

*Most mothers that come in with an abortion*, *they want the products out maybe in the next 2 minutes*, *they come with that mentality that now that it is lost*, *now there are some things remaining*, *I want maybe in four hours maximally to be out of hospital*. *And you know when you are giving misoprostol*, *it needs some time to work*. *(participant 35_doctor_referral hospital)*

In some instances, patients experienced failure to get a complete expulsion of products. Misoprostol failure may occur due to an incorrect dosing schedule which may be staff or patient influenced. Heavy workload and understaffing compromises close patient monitoring and effective delegation of duties to another HCP with inadvertent improper administration of the drug. Some of the staff taking over shifts may not follow the dosing frequency as required:

*Yes*, *you can talk to the mother at times like you may get a mother when you are going off*, *so you delegate someone who might not follow her up*. *Being one nurse on a ward*, *she might be busy and forget to go and see the mother taking the tablets*. *(participant 27_midwife_general hospital)*

On the other hand, a few patients are non-compliant to the dosing schedule due to peer influence from other patients on the ward. Patients may be reluctant or even pretend to take the misoprostol if they experience pain, or a HCP is rude. Pain relief should routinely be administered during treatment with misoprostol because patients experience uterine cramps and dilatation of the cervix during extrusion of the products of conception. However, analgesics were not given in some instances due to: lack of drug and supplies, the HCP being too busy or even a HCP refusing to administer the pain killer due to bad attitude, a sign of abortion stigma. One midwife’s view on patient peer influence:

*To some mothers it needs you to give her the drug right there … She may hide it and tell you that she took it and complain of not seeing any change*. *And you think like the drug is not working*. *… As you know*, *like when they are together in the gynae-ward … they are very many there*, *they can talk and talk*, *so peer influence*. *(participant 27_midwife_general hospital)*

A few doctors and clinical officers at the general hospitals are still inclined to surgical evacuation and therefore divert patients that would otherwise benefit from misoprostol management–probably because there is a financial incentive for doctors to perform surgical evacuations. A few women also held the belief that only surgical evacuation would remove all products:

*Though of course*, *with the beliefs that everyone has to be washed in the abdomen*, *even when the abortion is complete*, *they [patients] want to be washed in the abdomen*. *So*, *sometimes you have to explain to the mother and tell her that it is okay*, *it is now fine*, *you can … but still they want it*. *It is still a challenge*. *(participant 42_doctor_general hospital)*

Other misoprostol challenges cited were: the varying dosing schedules of misoprostol for different indications which confuse HCPs especially those with infrequent knowledge updates:

*If the midwife does not know the doses then that is a big challenge*. *I have told you that with MISO once you don’t know the doses you don’t know*. *If the mid wife is not well trained with the dosage*, *she may not be able to use it*. *(participant 17_midwife_health centre)*

Some HCPs report instances of misoprostol theft from the wards creating local drug shortages. There is also fear that a patient may use it to terminate a subsequent unintended pregnancy since she will have known how to use the drug:

*If the mother gets to know that in case*, *I get pregnant and this thing [misoprostol] can work*, *it may bring problems*. *She might go and buy it from somewhere and if she doesn’t want that pregnancy*, *she might take it because she knows how to do it*. *So*, *in that case*, *some who are very brilliant can-do abortions like that*. *(participant 27_midwife_general hospital)*

To address these challenges, the respondents’ main coping strategy was psychological support in terms of good patient rapport, continuous counseling and reassurance. This psychological support component was essential right from the time the patient presented at the health facility up until the time the patient returned to the community.

*We give them intra muscular tramadol or paracetamol tablets*, *some pain killers and we cover them then they get okay*. *(participant 23_midwife_general hospital)*

Other strategies to address the challenges included: provision of analgesics to reduce the pain and fever; proper risk assessment to ensure that patients given misoprostol fit the eligibility criteria; continuous patient monitoring to capture any complications early; and mentorship of other staff for attitude change towards PAC patients. One midwife’s view on patient’s close monitoring in the hospital:

… *like second trimester*, *we are observing these patients within a hospital setting*. *We tell them to report to the person on duty*. *… So that some caution is taken because in most cases*, *if that [side effect] is there*, *we have to give a remedy …*. *(participant 29_midwife_general hospital)*

#### The overstretched health system

At all health facilities, majority of the HCPs revealed heavy workload as the main challenge. With only one or two staff on a duty shift and many patients to care for, the staff get overwhelmed with job tasks which negatively affects the quality of care given:

*Of course*, *we are few doctors around*, *and we have a big clientele often times there may be delays in coming to access a mother who has been admitted as a doctor in a way it affects my performance*. *(participant 11_doctor_general hospital)*

Furthermore, facilities had inadequate space to operationalize monitoring of patients, with too few gynecological beds making patients sleep on the floor and shifting the task of monitoring to maternity patients which make PAC women face stigmatization or neglect:

… *sometimes we run short of beds … we give beds to those who have delivered because of prevention of infection to the baby*, *more than that one who has had an abortion*. *… of course*, *others feel neglected*. *(participant 1_midwife_health centre)*

Although health care at public facilities is supposed to be free, there are instances when essential drugs (antibiotics, analgesics) and supplies (gloves, intravenous fluids, blood, and cotton pads) are lacking and patients are requested to purchase them at nearby pharmacies. However, there are some patients who cannot even afford to buy these items, delaying institution of appropriate care:

… *sometimes … there are no gloves*, *… no IV fluids*, *you want to resuscitate*, *so you will have to first write the chit*, *you give the client to go and look for the things you need*. *(participant 26_midwife_health centre)*

Another challenge mentioned was the frequent staff rotations which deplete the number of skilled staff offering PAC services as described;

… *like us nurses and midwives*, *we are rotated*. *So*, *we are not static in one place*. *And then*, *at the same time when am not within source*, *and … or the people who are really active in that department are not at source*, *then*, *that is where the problem sets in*. *so they keep picking phones starting to look for where you are*, *where you could be*. *(participant 29_midwife_general hospital)*

Coping strategies for the work environment challenges included, staff commitment to work, working late hours, improvising when they lack supplies, patient involvement in their management, and having good teamwork. Albeit there are often insufficient number of staff at public facilities, the few skilled are competent in PAC:

*Now*, *it is a lot of work*, *actually sparing time for a patient to get PAC*, *ideally it is a team-work job and a lot of dedication … But all the same we handle*. *(participant 35_doctor_referral hospital)*

## Discussion

This study highlights HCPs’ perspectives on the use of misoprostol for second trimester PAC as well as the perceptions around task sharing. Importantly, midwives are described as competent in second trimester PAC albeit with the wish for back-up of a doctor. There is unanimous support for misoprostol, notwithstanding treatment-related and health system challenges experienced. The data informs policy formulation and successful implementation strategies at facility levels.

A key finding in our study is that both doctors and midwives are described as competent HCPs in using misoprostol for second trimester PAC. This is a contrast since second trimester PAC has mainly been the domain of doctors, stemming right from pre-service training [27]. Evidence from Uganda, Kenya, Senegal, and India shows that midwives can effectively and safely provide first trimester PAC with misoprostol [3,16,17,29,38]. While this prior experience could have primed the HCPs; training, mentorship and provision of guidelines are essential, for patient safety and HCP confidence [29].

A medical paradox here is, midwives being viewed as more competent than doctors in PAC, however a few doctors are still skeptical. This can be interpreted as reluctance of doctors to task share some roles especially when there’s expected monetary gain [39], and a fear of midwives’ encroachment in their territory. However, it may also be viewed as the hierarchical delineation of roles in medical practice, with doctors holding a supervisory role over midwives [7]. This agrees with previous evidence on workplace tensions between doctors and midwives in provision of maternal health care [40,41]. As midwives acquire additional knowledge and competences, their responsibilities at the workplace increase, and there is an expected discourse when midwives’ roles overlap with those of physicians. Since misoprostol is only used in uncomplicated cases [12], it is critical for HCPs to skillfully identify eligible patients and know when to revert to other treatment modalities [3]. The silver lining here is, midwives usually adhere to guidelines, and spend an appreciable time with patients on the ward [7]. During this period, they build rapport with the patients, monitor them, and can easily identify any deviations from the norm for consultation with the doctor or refer if the doctor is unavailable. In addition, midwives offer a wide range of services including: counseling, follow-up, and family planning [15]. In settings where doctors are scarce, task sharing some roles to midwives, helps increase access to quality PAC [3].

HCPs at all facility levels unanimously support misoprostol for second trimester PAC. While some doctors, were initially doubtful about its use for second trimester PAC, their participation in the ongoing trial changed the perception. Misoprostol is perceived as safe, effective, easy to administer, cheap, easy to store, and acceptable by patients and HCPs; and these findings agree with previous PAC studies [3,42,43]. Patients treated with misoprostol are also less likely to experience complications compared to surgical evacuation [42]. In essence, misoprostol saves resources like: reduced need for surgical facilities, less patient waiting time to treatment initiation, and opportunity for HCPs to multitask, improving their work efficiency [29]. Uganda’s policy guidelines on misoprostol for first trimester PAC are clear, disseminated, and the drug is available in government health facilities as an essential medicine [27]. This may ease introduction of misoprostol use for second trimester PAC, and abandon an outdated method (curettage) in favour of a safe and effective method.

A major challenge to misoprostol use were the known side effects. While side effects are expected [12,43], there is limited mention of this in previous qualitative studies [14,42]. HCPs devised coping strategies including: counseling, provision of analgesics, warm drinks, and coverings. Counseling is an essential PAC component [13]; that addresses patient expectations on procedures to follow, treatment success, duration of treatment and likely side effects. Effective counselling may enhance patient adherence to the treatment schedule despite experience of side effects.

Unlike first trimester PAC where patients receive misoprostol and return home [16,17], second trimester PAC patients are required to stay at the health facility until expulsion of all products [12], for prompt intervention in case of complications [42]. The prolonged time at the health facility is distressing especially for adolescents and women who have induced abortion, since they do not want to be identified with PAC [29]. The situation is further compounded by inadequate space at most health facilities, lack of prioritization for PAC patients and HCP stigma particularly in countries with restrictive abortion laws [44].

A few patients experience treatment failures mainly from inappropriate dosing schedules due to staff overload, with the midwives taking on majority of PAC roles [7,14,15]. Training, recruitment and retention of staff by government [3], and avoidance of staff rotations at facility level [29], are proposed solutions to address staff overload and enhance quality of care. At the facility level, HCPs encouraged patients to remind them the time for drug administration. While this may be unconventional, it depicts the innovativeness HCPs have to undertake to address the shortcomings in their work environments [7]. Patients’ involvement in their care provision, may serve three purposes: a) possibly increases treatment acceptability, b) enhances their psychological welfare and c) relieves some pressure off the HCPs.

Another reflection of the over stretched health system is the lack of essential supplies and drugs at some health facilities [7,28], yet patients may not afford to buy them from external pharmacies [7]. Stock-outs at the health facilities may reflect inadequate planning and forecasting and an erratic number of patients due to poor referral network [3,29]. At times facilities receive less stock than what they requested from the central national medical store [7]. Despite its resource intensiveness, a few doctors and clinical officers prefer surgical evacuation, motivators may be unwillingness to wait for the treatment outcome and monetary profit. Extortion of money from patients and theft of public drugs, to supplement the public servants’ meagre salaries, is a national concern and a special presidential team is assigned to investigate and identify culprits [45]. Therefore, access to medical products is essential for a functional productive health system [6].

### Research team and reflexivity

The researcher is a female Ugandan obstetrician and gynaecologist, with over ten years of research experience in post abortion care. As part of her PhD work, she’s currently conducting a RCT comparing the safety, effectiveness and acceptability outcomes of treatment for incomplete abortion using misoprostol when provided by midwives versus physicians in Uganda [31]. Since the main researcher had interacted with the HCPs on several occasions and the trial is still ongoing, two independent male interviewers, experienced in qualitative maternal health research conducted the interviews to reduce social desirability bias. The strength of our study is the in-depth understanding of the lived experiences for a wide range of HCPs with regards to professional cadre, level of health facility, years of work and PAC experience. Given the thick description of the study context, the results may be transferred to other similar settings where applicable.

## Conclusion

Misoprostol is highly favored as a uterine evacuation method for second trimester PAC of uncomplicated patients by HCPs. Given misoprostol’s ease of administration, trained midwives can competently manage these patients in a health facility setting albeit with a back-up of a doctor. Counseling, reassurance and involving patients in their care plans are proposed effective strategies that may address the challenges of side-effects and appropriate dosing. Still, an appropriate institutional and policy environment needs to be created for PAC in terms of staffing, workload, drugs and supplies, and physical space. Therefore, in similar settings with restrictive abortion laws and under-resourced health systems, task sharing second trimester PAC with midwives may be a feasible policy option that can increase access to care.

## Supporting information

S1 Table Respondents’ socio-demographic characteristics and work experience. (DOCX)

S1 File Interview guide for the study. (PDF)

## Abbreviations

HCPs: Health care providers
LMIC: Low- and middle-income countries
PAC: Post abortion care
RCT: Randomized controlled trial
WHO: World Health Organization
