# Supplementary material for: Exploring health care providers’ experiences of and perceptions towards the use of misoprostol for management of second trimester incomplete abortion in Central Uganda
Source: PLoS One. 2022 May 19;17(5):e0268812. doi: 10.1371/journal.pone.0268812 (PMC9119526; doi:10.1371/journal.pone.0268812)
Supplement: S1 Table — (DOCX) [file pone.0268812.s001.docx]

**S1 Table. Respondents' socio-demographic characteristics and work experience.**

| **Unique Number** | **Cadre** | **Sex** | **Age** | **Religion** | **Years practicing PAC** | **Years of clinical experience** | **Health facility level** |
| --- | --- | --- | --- | --- | --- | --- | --- |
| 1 | Midwife | Female | 33 | Anglican | 9 | 9 | Health centre IV |
| 2 | Midwife | Female | 29 | Catholic | 6 | 6 | Health centre IV |
| 3 | Medical Officer | Male | 60 | Catholic | 17 | 39 | Health centre IV |
| 4 | Midwife | Female | 32 | Anglican | 2 | 8 | General Hospital |
| 5 | Nursing Officer | Female | 27 | Anglican | 7 | 7 | General Hospital |
| 6 | Nursing Officer | Female | 40 | Moslem | 3 | 16 | General Hospital |
| 7 | Nursing Officer | Female | 40 | Anglican | 7 | 16 | General Hospital |
| 8 | Male | Male | 36 | Anglican | 9 | 9 | General Hospital |
| 9 | Midwife | Female | 36 | Anglican | 2 | 12 | General Hospital |
| 10 | Midwife | Female | 27 | Anglican | 2 | 5 | General Hospital |
| 11 | Medical Officer | Male | 47 | Anglican | 5 | 19 | General Hospital |
| 12 | Medical Officer | Male | 29 | Pentecostal | 4 | 5 | General Hospital |
| 13 | Midwife | Female | 38 | Anglican | 10 | 14 | Health centre IV |
| 14 | Medical Officer | Male | 38 | Anglican | 9 | 9 | Health centre IV |
| 15 | Midwife | Female | 25 | Catholic | 2 | 5 | General Hospital |
| 16 | Medical Officer | Male | 28 | Anglican | 1 | 1 | General Hospital |
| 17 | Midwife | Female | 37 | Moslem | 10 | 19 | Health centre IV |
| 18 | Midwife | Female | 29 | Catholic | 1 | 8 | Health centre IV |
| 19 | Midwife | Female | 48 | SDA | 4 | 22 | General Hospital |
| 20 | Midwife | Female | 28 | Catholic | 5 | 5 | General Hospital |
| 21 | Nursing Officer | Female | 50 | Catholic | 20 | 20 | General Hospital |
| 22 | Medical Officer | Male | 31 | Anglican | 6 | 6 | General Hospital |
| 23 | Midwife | Female | 42 | Anglican | 15 | 23 | General Hospital |
| 24 | Medical Officer | Male | 39 | Catholic | 2 | 5 | Health centre IV |
| 25 | Nursing Officer | Female | 36 | Catholic | 5 | 15 | Health centre IV |
| 26 | Midwife | Female | 35 | Catholic | 2 | 9 | Health centre IV |
| 27 | Midwife | Female | 59 | Catholic | 10 | 30 | General Hospital |
| 28 | Obstetrician & Gynaecologist | Male | 50 | Anglican | 20 | 20 | General Hospital |
| 29 | Nursing Officer | Female | 50 | Anglican | 12 | 25 | General Hospital |
| 30 | Nurse-midwife | Female | 51 | Anglican | 28 | 28 | General Hospital |
| 31 | Medical Officer | Male | 28 | Pentecostal | 2 | 2 | General Hospital |
| 32 | Nursing Officer | Female | 59 | SDA | 30 | 30 | General Hospital |
| 33 | Nurse-midwife | Female | 58 | Catholic | 10 | 24 | Referral Hospital |
| 34 | Midwife | Female | 52 | Catholic | 10 | 25 | Referral Hospital |
| 35 | Medical Officer | Male | 33 | Catholic | 7 | 7 | Referral Hospital |
| 36 | Nurse-midwife | Female | 48 | Catholic | 10 | 25 | Referral Hospital |
| 37 | Nurse-midwife | Female | 28 | Catholic | 5 | 5 | Referral Hospital |
| 39 | Obstetrician & Gynaecologist | Male | 50 | Catholic | 17 | 20 | Referral Hospital |
| 40 | Midwife | Female | 40 | Anglican | 6 | 16 | General Hospital |
| 41 | Nursing Officer | Female | 50 | Pentecostal | 27 | 27 | General Hospital |
| 42 | Medical Officer | Male | 31 | Catholic | 6 | 6 | General Hospital |
| 43 | Midwife | Female | 21 | Moslem | 1 | 2 | Referral Hospital |
| 44 | Medical Officer | Male | 30 | Pentecostal | 6 | 6 | Referral Hospital |
| 45 | Midwife | Female | 26 | Anglican | 2 | 4 | Referral Hospital |
| 46 | Nursing Officer | Female | 48 | Moslem | 6 | 21 | Referral Hospital |
| 47 | Medical Officer | Male | 33 | Catholic | 9 | 9 | Referral Hospital |
| 48 | Midwife | Female | 24 | Anglican | 5 | 5 | Referral Hospital |
| 49 | Medical Officer | Male | 40 | SDA | 2 | 7 | General Hospital |

*PAC – Post abortion care, SDA – Seventh day Adventist*
