# Supplementary material for: Exploring health care providers’ experiences of and perceptions towards the use of misoprostol for management of second trimester incomplete abortion in Central Uganda
Source: PLoS One. 2022 May 19;17(5):e0268812. doi: 10.1371/journal.pone.0268812 (PMC9119526; doi:10.1371/journal.pone.0268812)
Supplement: S1 File — (PDF) [file pone.0268812.s002.pdf]

S2 In-depth Interview Guide for the study.

Exploring health workers' experiences of and perceptions towards the use of misoprostol for management of incomplete mid trimester abortion

Introduction

- Thank the participant for accepting to be interviewed.
- Introduce yourself (facilitator) and the note taker and explain what each one will be doing.
- Share with the participant the aim of the interview – "to explore health workers' experiences of and perceptions towards the use of misoprostol for management of incomplete mid trimester abortion".
- Tell the participant the interview is expected to last one hour.
- Inform the participant that a tape recorder will be used to keep a record of the conversation. Assure the interviewee that the interview will be kept confidential.
- Explain that there is no right or wrong answer.
- Give the interviewee the consent form and allow them time to read it.
- Ask if there are any questions and clarify any queries raised at this point.
- If they accept to proceed with the interview, request the interviewee to sign the consent form.

<u>Interview characteristics</u>

Date of the interview: ……………………… Start time of the interview: ……………..

End time of the interview: ………………… Hours: …………………………………..

Health facility: ……………………………. Recorder: Yes □ No □

Identification number: …………………….

<u>Interviewee characteristics</u>

1. Socio-demographic characteristics

Sex: Male□ Female □ Religion: ……………………………….

Age: ……………………………………… Professional cadre: …………………….

2. Years in practice: …………………………

3. Years of work in post abortion care: ……..

<u>Meaning of abortion, incomplete abortion and post abortion care</u>

1) According to you, what is the meaning of abortion?

   Probe:

   - How can you differentiate a spontaneous abortion from an induced abortion?

2) How would you describe an incomplete abortion? Please explain.
3) Could you explain what you understand as post abortion care (PAC)? Probe for essential elements like emergency care, counseling, contraception, linkage to other reproductive health services and community involvement.

Provider's experiences on PAC

4) Describe to me what happens when a woman arrives at the health facility after having an abortion (be it spontaneous or induced).

   Probe:

   - Who is the main provider of PAC at your facility?
   - Who normally does the first clinical assessment of the patient at your facility?
   - Tell me the methods of uterine evacuation offered in the 2nd trimester?
   - Which other services are offered as part of PAC?
   - In your view, what are the characteristics of high quality Post Abortion Care (PAC)?

5) What types of contraceptives are offered to post abortion women at this health facility?

   Probe:

   - Any others?
   - Are they always available?
   - Are there any challenges in provision of post abortion contraception?

Provider's experiences on PAC Study

6) Having participated in the Randomized Controlled Trial (RCT), can you describe your experience on use of misoprostol for treatment of 2nd trimester incomplete abortion?

   Probe:

   - How effective do you think it is (able to empty the uterus of retained products within 24 hours)?
   - What is your view on its' safety as a method of uterine evacuation? Probe for its safety in terms of bleeding, infection, prolonged hospital stay (>48 hours).
   - Tell me about the benefits of using misoprostol for 2nd trimester PAC if any?
   - Any challenges faced?
   - How have you tried to overcome these challenges? Probe for explanation.

Provider's perceptions on PAC

7) How confident do you feel when providing PAC services?

   Probe:

   - Tell me more in terms of whether you feel that you received enough training in PAC? Probe for - who provided the training, how long was the training, how many trainings, and training on misoprostol for PAC.
   - What is your view on the level of staff at this facility that are trained to provide PAC?
   - If there's a patient in need of PAC services, and the available staff are not trained in PAC, please explain what happens.
   - Please explain how your work environment affects your time in delivering PAC services.

8) Tell me your view on midlevel providers (midwives, nurses, clinical officers) delivering PAC equally as safely and effectively as doctors.

Probe:

- In case MOH approves a policy on midlevel providers treating second trimester incomplete abortion using misoprostol, what are the likely barriers?
- How can they be overcome?

9) So, how do health workers feel about the women who have self-induced an abortion? Probe for stigma.

10) Do you have any questions or comments?

Closing

- Thank the interviewee for their participation.
- State that the discussion findings will be kept confidential.
- Provide the participant the reimbursement cost.
